# Integration analysis of lncRNA and mRNA expression data identifies DOCK4 as a potential biomarker for elderly osteoporosis

**DOI:** 10.1186/s12920-024-01837-3

**Published:** 2024-03-05

**Authors:** Chengai Wu, Chao Wang, Bin Xiao, Shan Li, Yueyang Sheng, Qianqian Wang, Jianfeng Tao, Yanzhuo Zhang, Xu Jiang

**Affiliations:** 1 Department of Molecular Orthopaedics, National Center for Orthopaedics, Beijing Research Institute of Traumatology and Orthopaedics, Beijing Jishuitan Hospital, Capital Medical University, Beijing, 100035 China; 2 Department of Spine Surgery, Beijing Jishuitan Hospital, Capital Medical University, Beijing, 100035 China; 3 Department of Orthopaedics, Beijing Jishuitan Hospital, Capital Medical University, No. 31, Xinjiekou East Street, Xicheng District, Beijing, 100035 China

**Keywords:** Elderly osteoporosis, Diagnostic, DOCK4, lncRNA, miRNA

## Abstract

**Background:**

We aimed to identify some potential biomarkers for elderly osteoporosis (OP) by integral analysis of lncRNA and mRNA expression data.

**Methods:**

A total of 8 OP cases and 5 healthy participants were included in the study. Fasting peripheral venous blood samples were collected from individuals, and total RNA was extracted. RNA-seq library was prepared and sequenced on the Illumina HiSeq platform. Differential gene expression analysis was performed using “DESeq2” package in R language. Functional enrichment analysis was conducted using the “clusterProfiler” package, and the cis- and trans-regulatory relationships between lncRNA and target mRNA were analyzed by the lncTar software. A protein-protein interaction (PPI) network was constructed using the STRING database, and hub genes were identified through the MCODE plugin in Cytoscape.

**Results:**

We identified 897 differentially expressed lncRNAs (DELs) and 1366 differentially expressed genes (DEGs) between normal and OP samples. After co-expression network analysis and cis-trans regulatory genes analysis, we identified 69 candidate genes regulated by lncRNAs. Then we further screened 7 genes after PPI analysis. The target gene DOCK4, trans-regulated by two lncRNAs, was found to be significantly upregulated in OP samples. Additionally, 4 miRNAs were identified as potential regulators of DOCK4. The potential diagnostic value of DOCK4 and its two trans-regulatory lncRNAs was supported by ROC analysis, indicating their potential as biomarkers for OP.

**Conclusion:**

DOCK4 is a potential biomarker for elderly osteoporosis diagnostic. It is identified to be regulated by two lncRNAs and four miRNAs.

**Supplementary Information:**

The online version contains supplementary material available at 10.1186/s12920-024-01837-3.

## Introduction

Osteoporosis (OP) is a common bone disease characterized by the weakening of bones, leading to increased fragility and susceptibility to fractures [1]. This condition primarily occurs due to a decrease in bone mineral density (BMD) and mass [2]. Aging is a significant factor, as bone density naturally declines with age. Other contributing factors include genetics, gender (more common in women), hormonal changes (especially a decrease in estrogen levels in postmenopausal women), lifestyle choices, inadequate nutrition, lack of physical activity, and certain medical conditions or medications [3]. Therapeutic strategies aim to increase bone density, reduce fracture risk, and improve patients' quality of life. Treatment may involve medications, such as bisphosphonates, calcium and vitamin D supplements, regular weight-bearing exercise, smoking cessation, and limiting alcohol consumption [4]. Early diagnosis and intervention are crucial in effectively managing OP and reducing the associated risks of fractures [5]. The global aging population is increasing, with age being a significant contributor to the risk of developing OP [6]. Data from the seventh national population census conducted in China in 2020 shows that the population aged 60 and above is 264 million, accounting for 18.7% of the total population. It is projected that by 2050, the elderly population in China aged 60 and above will exceed 400 million [7]. In other Asian countries such as Korea [8] and India [9], 60-year-old is also an age in danger for OP.

Long non-coding RNAs (lncRNAs) are a class of RNA molecules that do not code for proteins but play important roles in various cellular processes, including growth, differentiation, etc [10]. They are typically longer than 200 nucleotides with a similar basic structure to mRNAs, including 5' and 3' ends, and can be spliced, polyadenylated, and capped. They are transcribed from various regions of the genome [11]. The evidence suggests that lncRNAs are at least four times longer than sequences of coding RNA [12]. LncRNAs are involved in a wide range of cellular processes, including transcriptional regulation, chromatin modification, epigenetic regulation, post-transcriptional processing, and cellular signaling [13]. They can act as molecular scaffolds, guides, decoys, or as direct regulators of gene expression [14]. By employing high-throughput sequencing and conducting bioinformatics analyses, an increasing body of evidence has demonstrated that numerous lncRNAs exhibit abnormal expression patterns in individuals with OP in comparison to those without the condition, suggesting that lncRNAs likely play a role in the onset and progression of osteoporosis [15]. LncRNAs show great potential in treating OP, and they have been utilized as biomarkers in many diseases, but they are lacking as such in OP [16].

Integrating lncRNA and mRNA data in the study of OP is highly advantageous for revealing more comprehensive molecular mechanisms, identifying potential biomarkers, and predicting disease progression and treatment responses.

## Materials and methods

### Research subjects

#### Ethical statements

This research has been approved by the Ethics Committee of Beijing Jishuitan Hospital (Ethics Approval Number: 201611-03). All research participants voluntarily joined this study and signed an informed consent form.

From November 2019 to August 2020, individuals diagnosed with OP and participants in health check-ups at Beijing Jishuitan Hospital were selected as the study subjects (Table 1) according to the diagnosis criteria of: Dual-Energy X-ray Absorptiometry (DXA) measurement of bone density with values below 1 standard deviation (SD) from the peak bone mass of healthy adults of the same gender and race was considered normal (T-score ≥ -1.0 SD); a decrease of 1 to 2.5 SD was categorized as low bone mass or bone loss (-2.5 SD < T-score < -1.0 SD); a decrease equal to or greater than 2.5 SD was diagnosed as osteoporosis (T-score ≤ -2.5 SD); when the degree of decrease meets the diagnostic criteria for osteoporosis and was accompanied by one or more fractures, it was classified as severe osteoporosis [17]. Inclusion criteria: The cases must meet the diagnostic criteria for OP; both OP cases and health check-up participants must be aged 60 or older. Exclusion criteria for cases include: (1) secondary or idiopathic OP patients; (2) patients with diabetes, hyperthyroidism, hyperparathyroidism, primary or secondary adrenal dysfunction, malignant tumors, cardiovascular or cerebrovascular diseases; (3) individuals who had taken alendronate sodium, calcium supplements, or other medications affecting bone metabolism for more than 3 months before diagnosis; (4) those with ankylosing spondylitis and rheumatoid arthritis; (5) individuals with a history of long-term immunosuppressant use. Exclusion criteria for healthy participants, in addition to the above, also involve excluding individuals with OP, those affected by metabolic bone diseases, and those undergoing treatment with bone metabolism-related medications. A total of 8 OP cases and 5 healthy participants were included in the study. The patients consisted of 1 male and 7 females with an average age of (72.4±4.2) years; among the 5 healthy individuals who underwent physical examinations without measuring Osteocalcin and 25-hydroxyvitamin D levels, there was 1 male and 4 females with an average age of (68.2±5.7) years. Male samples were hard to collect because gender was known to be associated with OP and the incidence rate of females were much higher than males [18].

**Table 1:** Clinical information of study subjects

PID	Group	Gender	Age	Osteocalcin (ng/ml)	25-hydroxyvitamin D (ng/ml)
OP01	OP	F	67	8.5	41.45
OP02	OP	F	79	8.12	27.23
OP03	OP	F	82	7.94	13.54
OP04	OP	F	61	13.3	58.71
OP05	OP	F	73	16.79	14.27
OP06	OP	M	77	16.62	20.89
OP07	OP	F	69	32.76	27.41
OP08	OP	F	71	15.33	11.83
Nor01	Normal	M	Average age: 68.24±5.7		
Nor02	Normal	F			
Nor03	Normal	F			
Nor04	Normal	F			
Nor05	Normal	F			

Furthermore, we obtained GSE230665 from the Gene Expression Omnibus (GEO) database (https://www.ncbi.nlm.nih.gov/geo/) for use as a validation set, comprising 3 normal samples and 12 OP patient samples. Then we applied GSE35959 as an independent dataset for the target gene validation containing 5 OP samples and 9 normal samples. Additionally, GSE64433 containing 3 OP samples and 3 control samples was used for predicting miRNA-mRNA regulatory relationships.

### Samples collection

Eight OP patients and five healthy individuals underwent fasting peripheral venous blood extraction in the early morning, utilizing ethylenediaminetetraacetic acid (EDTA) anticoagulant tubes for collection. Subsequently, the collected blood was gently inverted and mixed approximately 10 times to ensure proper blending of the anticoagulant with the blood.

### Transcriptome sequencing

Total RNA extraction was performed using the Trizol reagent kit (Invitrogen Corporation, Waltham, USA) as follows: (1) One 15 ml tube was taken, and 6 ml of Trizol and 2 ml of fresh blood (Trizol to blood volume ratio of 3:1) were added. (2) The sample was vigorously shaken for 1-2 minutes until completely dissolved. (3) Incubation at room temperature for 5 minutes was conducted to ensure complete degradation of nuclear proteins. (4) The tube was sealed, flash-frozen in liquid nitrogen, and stored at -80 ℃.

The quality of total RNA samples was assessed using an Agilent 2100 BioAnalyzer (Agilent Technologies, Inc., Santa Clara, USA) and Qubit Fluorometer (Invitrogen, USA). The criteria for quality assurance included: (1) RNA Integrity Number (RIN) > 7.0; (2) Characteristic features in the electrophoretic profiles of 28S rRNA and 18S rRNA: the brightness of 28S rRNA should be greater than that of 18S rRNA, with a 28S:18S ratio exceeding 1.8.

The preparation and sequencing of the RNA-seq library were carried out by CapitalBio Technology Company (Beijing, China). KAPA library quantification kit (KAPA Biosystems, Inc., South Africa) and Agilent 2100 Bioanalyzer were used for library quantification. After verification through quantitative reverse transcription polymerase chain reaction (RT-qPCR), the library was sequenced on the Illumina HiSeq sequencer (Illumina, Inc., San Diego, USA).

### Differential expression analysis

In this study, we performed all statistical analyses by R language (4.1.0). The “DESeq2” package was utilized for the screening of differentially expressed genes (DEGs) or differentially expressed lncRNAs (DELs), with the criteria of an absolute Log_2_FC value greater than 1 and a *p*-value less than 0.05. Genes with log_2_FC ≤ 1 were considered upregulated, while those with log_2_FC ≤ -1 were considered downregulated. Volcano plots were generated using the “ggplot2” package.

### Functional enrichment analysis

Using the identified DEGs, we applied the "clusterProfiler" package [19] to perform functional enrichment analysis, encompassing Gene Ontology (GO) terms (Biological Process (BP), Molecular Function (MF), and Cellular Component (CC)) and Kyoto Encyclopedia of Genes and Genomes (KEGG) pathways. The significance threshold for enriched terms and pathways was set at a *p*-value < 0.05.

### The prediction of target genes regulated by both cis-acting and trans-acting mechanisms of DELs

Using the lncTar software, we predicted whether there is targeted regulation, thus conducting a comprehensive analysis of the cis- and trans-regulatory relationships between lncRNA and target mRNA. This analysis involved integrating expression data, relative positioning, and sequence information at three levels to elucidate the potential regulatory interactions between lncRNAs and their target mRNAs.

### Protein-protein-interaction (PPI) network

Utilizing the online database STRING (https://string-db.org/), a PPI network was constructed. Subsequently, and the established network structure was subjected to the MCODE plugin algorithm in Cytoscape to identify hub genes.

### Statistical analyses

For continuous variables, Student's t-test was employed to group and compare normally distributed variables. Receiver Operating Characteristic (ROC) curve analysis was conducted to assess the diagnostic efficacy of the included diagnostic biomarkers. A *p*-value less than 0.05 was considered indicative of statistically significant analyses.

## Results

### Identification of DEGs and DELs between normal samples and OP samples

**Fig. 1:**
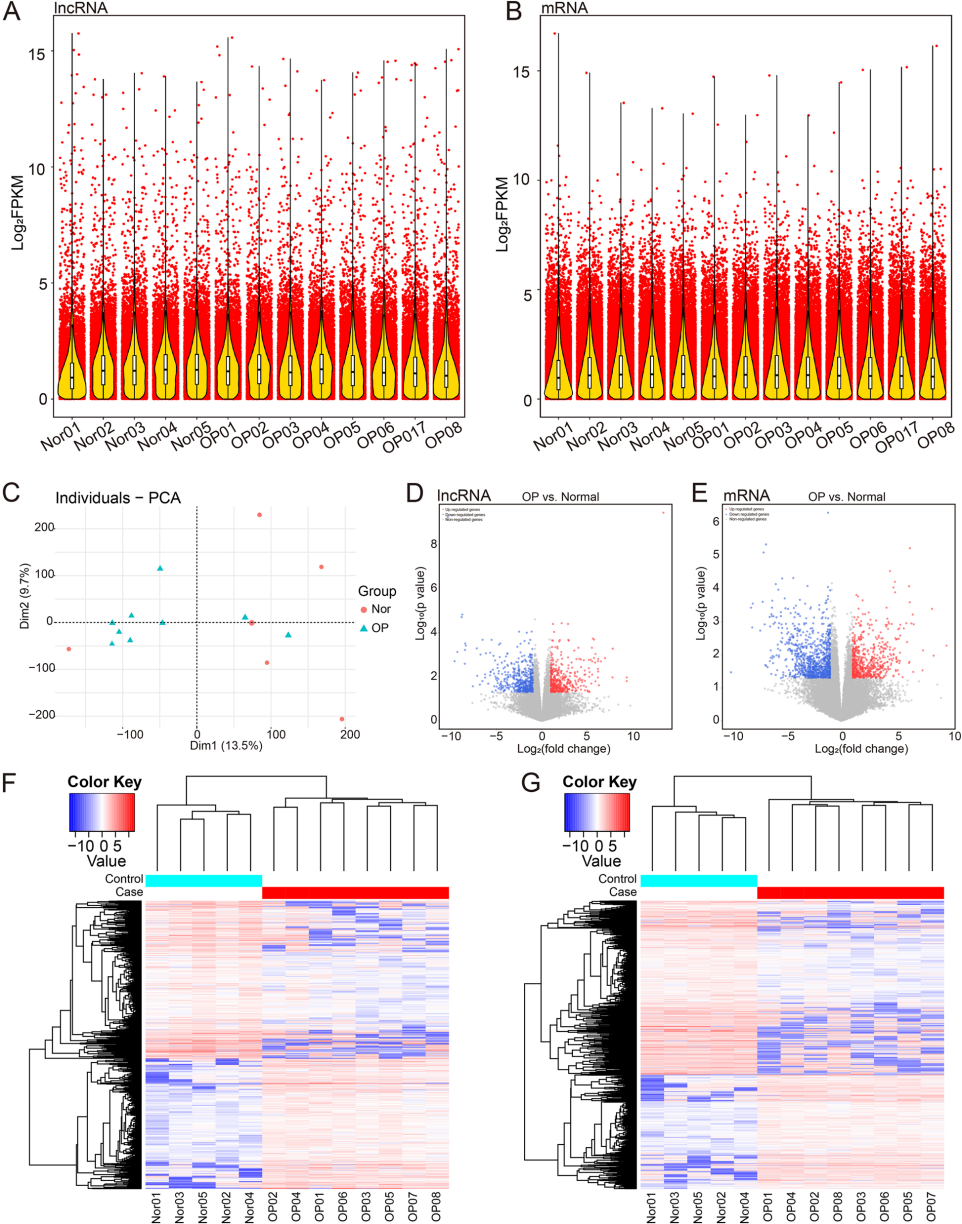
Identification of DEGs and DELs between normal samples and OP samples. A-B FPKM of lncRNA and mRNA data, respectively. C PCA of OP and normal samples based on transcriptome data. D-E Differential expression analysis of lncRNAs and mRNAs between OP and normal samples. F-G Heatmap of differential expression analysis of lncRNAs and mRNAs between OP and normal samples, respectively

The IDs of known lncRNAs and novel lncRNAs of the normal group and OP group were obtained from the transcriptome data. Subsequently, based on these IDs, the expression information for lncRNAs and mRNAs was extracted separately from the results list of transcriptome expression analysis in the total gene, displayed in Fragments Per Kilo bases per Million fragments (FPKM) (Fig. 1A-B). Then principal component analysis (PCA) was performed on normal samples and OP samples according to the transcriptome data, and the samples of different groups could be mostly divided into two parts (Fig. 1C).

By differential expression analysis, 897 DELs and 1366 DEGs between normal and OP samples were identified. There were 406 upregulated and 491 downregulated DELs in OP samples compared to normal samples (Fig. 1D). A total of 536 upregulated and 830 downregulated DEGs were identified in OP samples (Fig. 1E). The levels of lncRNAs and mRNAs were different between the two groups (Fig. 1F-G). Detailed results of differential expression analysis were shown in Table S1.

### The establishment of a co-expression network between lncRNA and mRNA and the determination of candidate genes and lncRNAs

**Fig. 2:**
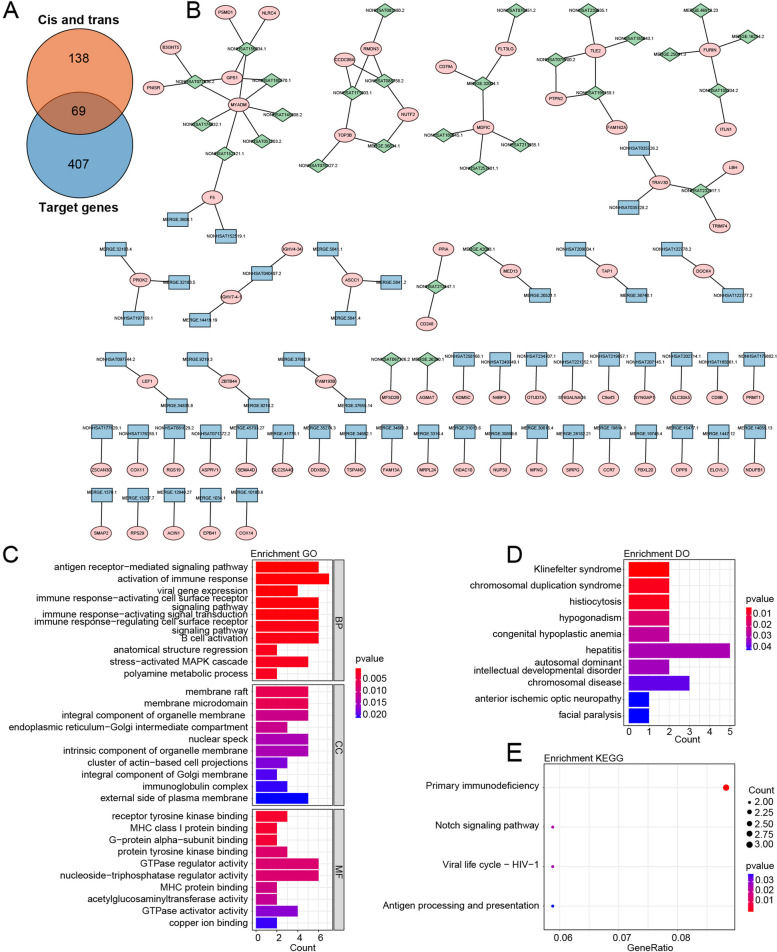
Determination of candidate genes and lncRNAs. A Intersection of target genes screened by co-expression analysis and DEGs under cis or trans regulation of DELs. B Candidate genes and their corresponding lncRNAs. Green represents cis regulation, and blue represents trans regulation. C-E Top 10 enriched GO terms, DO and KEGG pathways of 69 candidate genes

LncRNAs and mRNAs are closely related to each other. In this study, the co-expression correlation between DELs and DEGs among samples was analyzed using the Pearson correlation coefficient method. A total of 476 DEGs were selected as differential target genes, based on a Pearson correlation coefficient absolute value greater than 0.99 and a *p*-value less than 0.05. Among the DELs screened by the co-expression analysis, 99 corresponding target genes under cis-regulation (Fig. S1) and 121 genes under trans-regulation (Fig. S2) were predicted (Table S2). After that, we took the intersection of the predicted 99 cis-regulated genes and 121 trans-regulated genes with the 476 differential target genes, resulting in 69 commonly shared genes as the candidate genes that met the correlation threshold (Fig. 2A). The 69 candidate genes were combined with their corresponding lncRNAs to construct a gene regulatory network, and visualization was performed using Cytoscape 3.3 (Fig. 2B).

Subsequently, we conducted GO, KEGG, and DO enrichment analyses for the 69 candidate genes. The results revealed significant enrichment in GO terms such as “antigen receptor-mediated signaling pathway”, “membrane raft”, and “receptor tyrosine kinase binding” (Fig. 2C), as well as in DO terms like “Klinefelter syndrome” (Fig. 2D) and the KEGG pathway “Primary immunodeficiency” (Fig. 2E). Comprehensive list of enriched results was shown in Table S3.

### Determination of target gene DOCK4

**Fig. 3:**
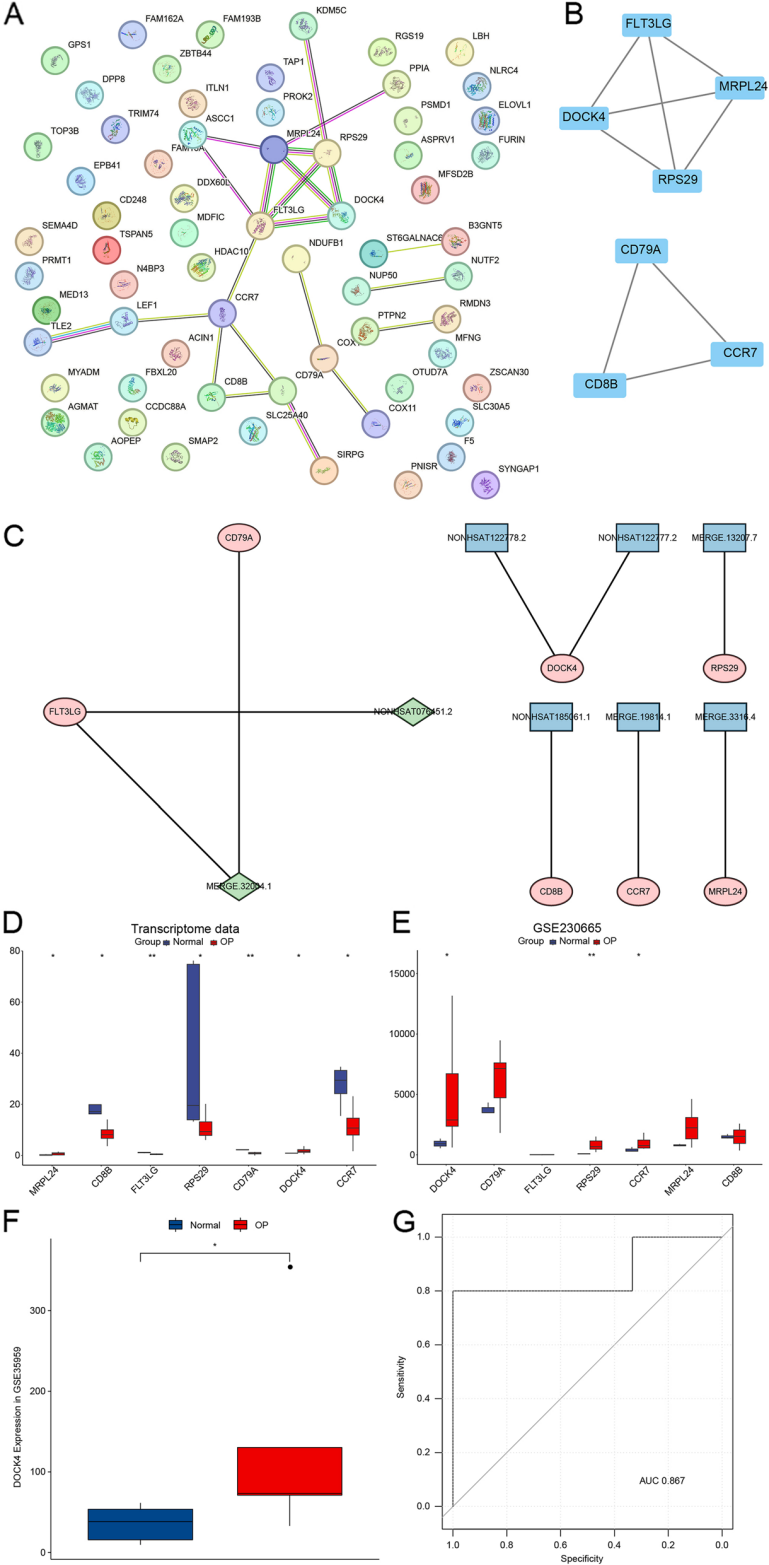
Determination of target gene DOCK4. A PPI network of 69 candidate genes. B Hub genes network screened by MCODE. C The relationship between 7 hub genes and their corresponding lncRNAs. D-E Expression levels of 7 hub genes in transcriptome data and GSE230665 dataset, respectively. F Expression of DOCK4 in OP and normal samples based on GSE35959 dataset. G ROC analysis using GSE35959 dataset

To screen potential biomarkers for OP, we performed PPI analysis on the 69 candidate genes using online STRING database, resulting in a PPI network with a total of 66 nodes and 22 edges. The average node degree value was 0.667, and the PPI enrichment *p*-value was 0.379 (Fig. 3A, Table S4). Using the cytoHubba plugin's MCODE algorithm, we identified two hub gene networks (Fig. 3B), consisting of 7 genes. Correspondingly, there were 8 cis-regulatory and trans-regulatory lncRNAs associated with these hub gene networks (Fig. 3C). Then we validated these 7 genes’ expression levels in our transcriptome data (Fig. 3D) and GSE230665 dataset (Fig. 3E). DOCK4 expressed significantly higher in OP than normal samples in both data. Then the expression level of DOCK4 was validated in GSE35959 showing significantly higher level in OP group (Fig. 3F). Area Under the Curve (AUC) value ROC curve was 0.867, suggesting DOCK4’s capability to distinguish OP and normal samples (Fig. 3G). Hence, DOCK4 was selected as our target gene, trans-regulated by two lncRNAs NONHSAT122778.2 and NONHSAT122777.2.

### Regulation of miRNAs targeting DOCK4

**Fig. 4:**
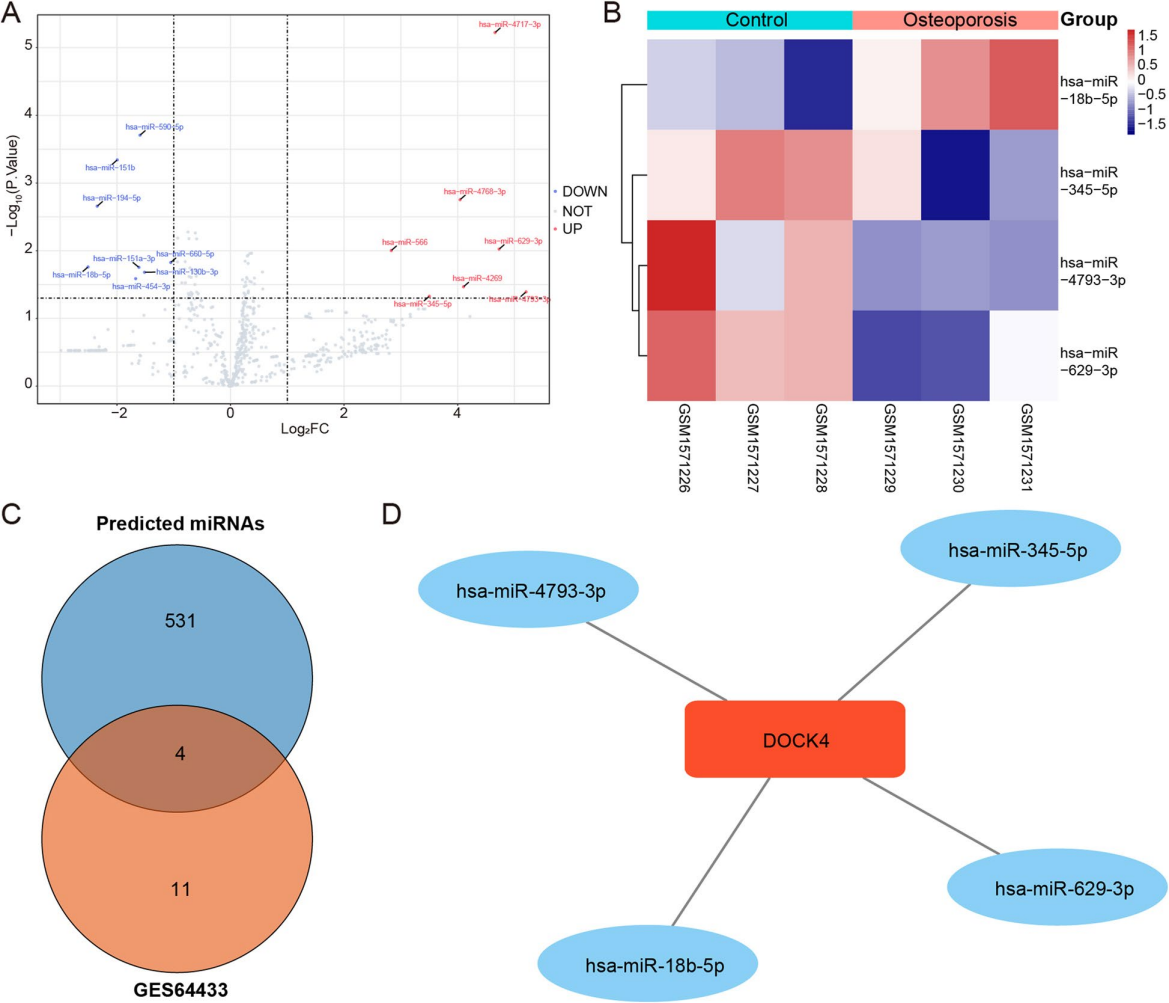
Regulation of miRNAs targeting DOCK4. A-B Volcano plot and heatmap of differential expression analysis of miRNAs between OP and control groups. C Intersection of predicted miRNAs targeting DOCK4 and DEmiRNAs between OP and control groups. D Network of DOCK4 and its regulatory miRNAs

Both miRNAs and lncRNAs are involved in post-transcriptional regulation. MiRNAs typically target mRNAs for degradation or translational repression, while lncRNAs can modulate gene expression at various levels. Investigating how miRNAs and lncRNAs jointly regulate mRNA targets can provide a more comprehensive understanding of gene expression control. We employed the TargetScan database (https://www.targetscan.org/vert_80/) to predict miRNAs targeting the DOCK4 gene, identifying a total of 661 target miRNAs. Differential analysis was performed on GSE64433 dataset, and using a threshold of |log_2_FC| > 1 and *P*-value < 0.05. We identified 15 differentially expressed miRNAs (DEmiRNAs) between the OP group and the control group (Fig. 4A). The expression levels of DEmiRNAs were distinct between the two groups (Fig. 4B). After intersecting these 15 DEmiRNAs with the 661 Target miRNAs, 4 commonly shared miRNAs were yielded (Fig. 4C), and hsa-miR-4793-3p, hsa-miR-18b-5p, hsa-miR-629-3p, and hsa-miR-345-5p were identified as regulators of DOCK4 (Fig. 4D).

### Potential diagnostic value of DOCK4 and its two trans-regulatory lncRNAs

**Fig. 5:**
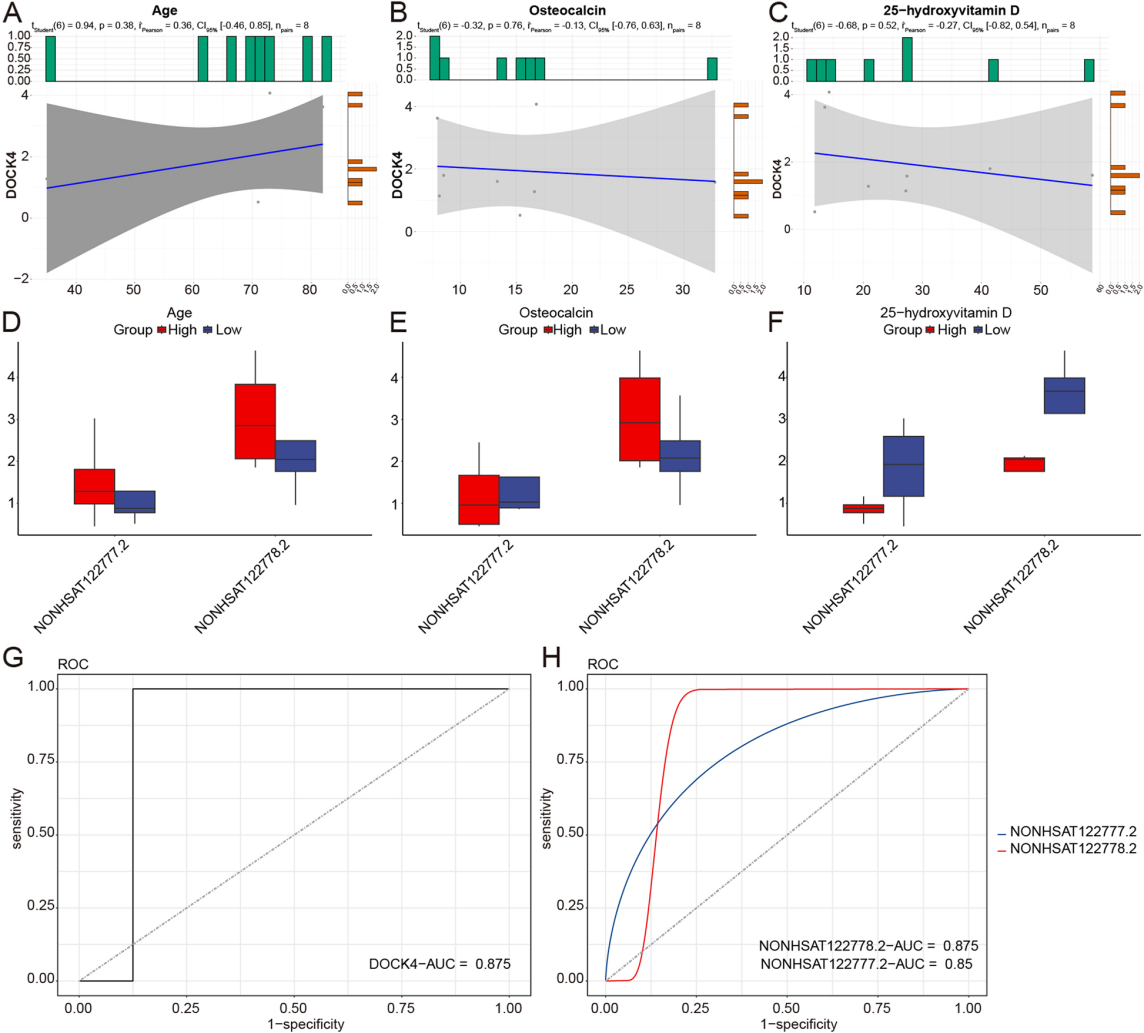
Potential diagnostic value of DOCK4 and its two trans-regulatory lncRNAs. A-C Correlation of DOCK4 expression and age, osteocalcin, as well as 25-hydroxyvitamin D. D-F Correlation between expression of lncRNAs regulating DOCK4 and age, osteocalcin, as well as 25-hydroxyvitamin D. G-H ROC analysis based on our sequencing data and GSE230665 dataset, respectively

Next, we investigated the correlation of DOCK4 with age, osteocalcin, and 25-hydroxyvitamin D, along with two corresponding lncRNAs. Our findings revealed a non-significant negative correlation between DOCK4 and osteocalcin as well as 25-hydroxyvitamin D, while a positive correlation was observed with age (Fig. 5A-C). In the case of the two corresponding lncRNAs, the expression levels of both lncRNAs were higher in the older group. NONHSAT122778.2 expressed higher in the group with higher osteocalcin level. Both lncRNAs regulating DOCK4 expressed relatively higher in groups with lower 25-hydroxyvitamin D levels (Fig. 5D-F).

To confirm the diagnostic value of DOCK4 and the two corresponding lncRNAs, we performed ROC analysis based on our sequencing data and GSE230665 dataset, showing that AUC values for DOCK4 and the two corresponding lncRNAs were greater than 0.5, indicating a potential diagnostic capability (Fig. 5G-H).

## Discussion

As individuals surpass the age of 50, the occurrence of fragility fractures steadily rises. The global trend of population aging is anticipated to contribute to a higher percentage of the world's population experiencing OP and fractures [20]. Focusing on elderly OP, we sequenced the transcriptome of 8 patients and 5 normal participants, obtaining the sequence of mRNA and lncRNA. By integral analysis of mRNA and lncRNA, we identified a gene DOCK4 potentially diagnostically valuable for OP trans-regulated by lncRNAs NONHSAT122778.2 and NONHSAT122777.2.

DOCK4, namely dedicator of cytokinesis protein 4, belongs to DOCK protein family including 11 members, from 1 to 11 [21]. The members are divided into 4 subgroups A, B, C, and D based on their sequences and domains [22]. DOCK4, a member of the DOCK-B subgroup, serves as Rac1 guanine nucleotide exchange factor [23]. DOCK4 has been proven to be a potential marker for bone metastasis in early breast cancer. DOCK4 was upregulated in bone-homing bone metastasis (BM)1 cells which was able to enhance invasive ability. Interestingly, elevated expression of DOCK4 did not correlate with metastasis to sites outside the skeletal system [24], showing the close relationship between DOCK4 and bone. Upon activation of RhoG, DOCK4 could form a complex initiating Rac activation to enhance lamellipodia formation and facilitate cell migration [25]. When we identified 69 candidate genes, functional enrichment analyses were performed on them, indicating to be enriched on immune-related terms or pathways such as activation of immune response and primary immunodefiency. DOCK4 has been identified to be a biomarker in stomach adenocarcinoma related to immune infiltration [26], so is in colon adenocarcinoma [27]. We noticed that the important biomarker osteocalcin encoding gene BGLAP for OP was not identified as a DEG between OP and healthy group due to the strict threshold we applied. If the standard of an absolute value of log_2_FC greater than 0.6, corresponding to a fold change of 1.5 was applied, BGLAP would have been identified.

In our study, 897 DELs were identified between OP and normal samples. After co-expression analysis, we screen 99 genes under cis-regulation and 121 genes under trans-regulation by DELs. Our target gene DOCK4 was trans-regulated by NONHSAT122778.2 and NONHSAT122777.2. LncRNAs have been found to modulate proliferation and apoptosis of osteoblasts in OP [28]. We deduce that NONHSAT122778.2 and NONHSAT122777.2 may influence osteoblasts or osteoclasts not only by regulating DOCK4, but also possibly control other related genes. Also, we explored the regulation of miRNAs for DOCK4 and found four miRNAs potentially modulating DOCK4. A miRNA miR-320a is overexpressed in OP bone tissue and it regulates expression of osteoblast-related genes [29]. MiRNAs play vital roles in bone metastasis in a lot of cancers, such as breast cancer, prostate cancer, lung cancer and renal cell carcinoma [30], indicating their key functions in bone. LncRNA and miRNA can work together to control DOCK4’s expression. LncRNA EBLN3P interacts directly with miR-144-3p, reducing the binding of miR-144-3p to the 3ʹ region of DOCK4 [31]. LncRNA AC073284.4 is able to inhibit epithelial-mesenchymal transition (EMT) and migration in breast cancer cells through the regulation of the miR‐18b‐5p/DOCK4 axis [32]. Similar interaction of lncRNA and miRNA might also exist in DOCK4 functioning of OP.

Our study identifies DOCK4 as a potential marker for OP diagnosis. However, it’s a pity that correlation of DOCK4 with age, osteocalcin, and 25-hydroxyvitamin D were not significant, probably due to the limited number of samples. We will try to collect more samples for deeper investigating clinical relation with DOCK4. Furthermore, the treatment value exploration of DOCK4 in OP will also be in plan.

Despite the successful identification of DOCK4 as a potential biomarker for osteoporosis (OP) through the integration analysis of lncRNA and mRNA expression data, and the potential regulatory mechanisms involving lncRNAs and miRNAs, the limited sample size in our study is a significant limitation. To enhance the validation of DOCK4 as a diagnostic marker for OP, we plan to expand the sample size in future research, encompassing a more diverse population in terms of gender, age, and geographical location, as well as including a larger control group. This will provide a more robust basis for understanding the role of DOCK4 in the pathogenesis of OP and its generalizability as a diagnostic marker. Furthermore, we aim to investigate the biological functions of DOCK4 in OP more thoroughly, including its specific contributions to bone metabolism and how it influences bone density through the regulation of cellular processes such as signaling, proliferation, and apoptosis. We will also explore the interplay between DOCK4 and other genes and pathways implicated in OP to better understand the complex molecular mechanisms involved. In terms of clinical implications, we plan to conduct prospective studies to assess the utility of DOCK4 as a tool for early diagnosis and monitoring disease progression. This will involve the development and validation of diagnostic tools based on DOCK4, such as blood tests, and evaluating their potential for personalized medicine approaches. Lastly, we will explore DOCK4 as a potential therapeutic target, considering drug interventions or gene therapies that could modulate its expression to mitigate the progression of OP and improve patient outcomes. These studies will contribute to the development of novel preventive, diagnostic, and therapeutic strategies for OP, potentially leading to improved patient care and quality of life.

## Conclusions

To summarize, the study utilized transcriptome sequencing and bioinformatics analysis to reveal a network of genes and lncRNAs involved in OP pathogenesis. DOCK4 was found to be significantly upregulated in OP patients. This research has identified DOCK4 as a potential diagnostic marker for osteoporosis, regulated by lncRNAs NONHSAT122778.2 and NONHSAT122777.2 and four miRNAs. Despite limited clinical correlations, the study provides a basis for future research with larger cohorts and more comprehensive clinical assessments, potentially leading to novel diagnostic and therapeutic strategies for OP.

### Supplementary Information

**Additional file 1:** **Fig. S1.** Network of DEGs cis-regulated by DELs.

**Additional file 2:** **Fig. S2.** Network of DEGs trans-regulated by DELs.

**Additional file 3:** **Table S1.** Detailed results of differential expression analysis.

**Additional file 4:** **Table S2.** Detailed results of cis and trans regulated genes.

**Additional file 5:** **Table S3.** Detailed information of functional enrichment analysis.

**Additional file 6:** **Table S4.** PPI network of 69 candidate genes.

## Data Availability

GSE230665 and GSE64433 utilized and analyzed during this study were available in the Gene Expression Omnibus (GEO) database (https://www.ncbi.nlm.nih.gov/geo/).

## Abbreviations

BM: Bone metastasis
BMD: Bone mineral density
DEGs: Differentially expressed genes
DELs: Differentially expressed lncRNAs
FPKM: Fragments Per Kilo bases per Million fragments
GEO: Gene Expression Omnibus
KEGG: Kyoto Encyclopedia of Genes and Genomes
lncRNAs: Long non-coding RNAs
OP: Osteoporosis
PPI: Protein-protein-interaction
PCA: Principal component analysis
EMT: Epithelial-mesenchymal transition
