# Supplementary figures and images for: Integration analysis of lncRNA and mRNA expression data identifies DOCK4 as a potential biomarker for elderly osteoporosis
Source: BMC Med Genomics. 2024 Mar 5;17:70. doi: 10.1186/s12920-024-01837-3 (PMC10916189; doi:10.1186/s12920-024-01837-3)

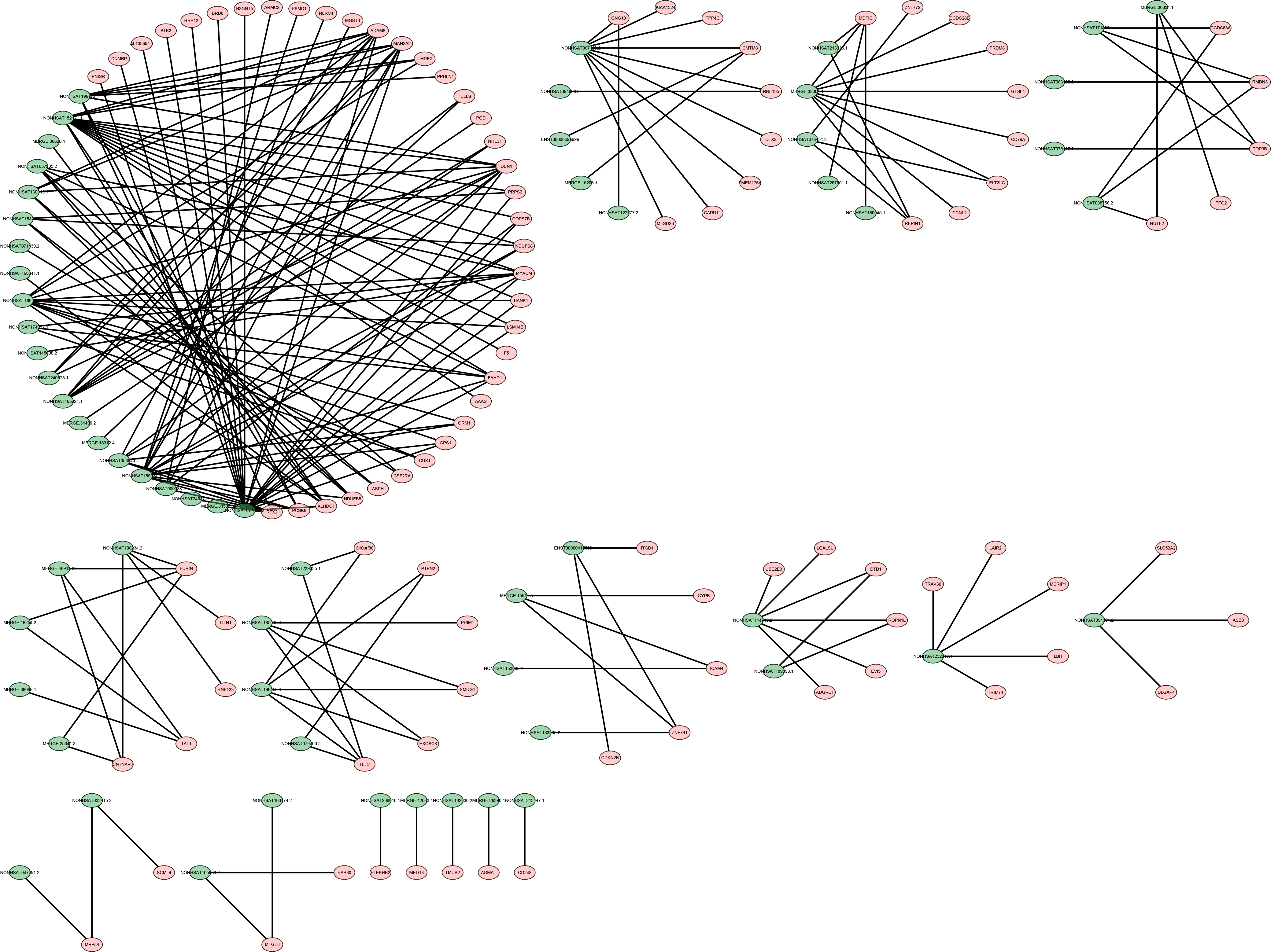

Supplement: Supplementary file 1 — Additional file 1: Fig. S1. Network of DEGs cis-regulated by DELs. [file 12920_2024_1837_MOESM1_ESM.jpg]

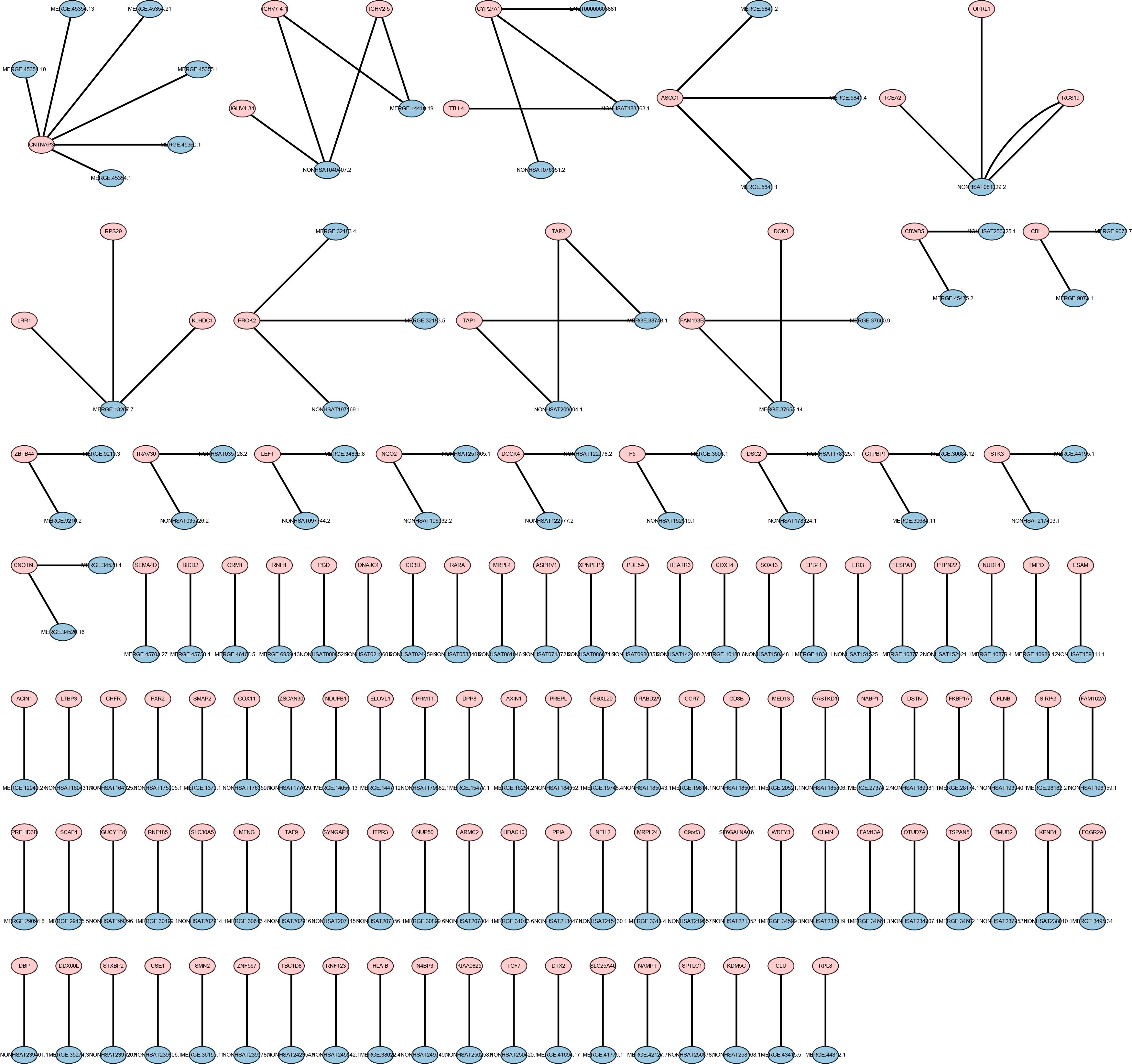

Supplement: Supplementary file 2 — Additional file 2: Fig. S2. Network of DEGs trans-regulated by DELs. [file 12920_2024_1837_MOESM2_ESM.jpg]
